# Supplementary material for: Oligonol®, an Oligomerized Polyphenol from Litchi chinensis, Enhances Branched-Chain Amino Acid Transportation and Catabolism to Alleviate Sarcopenia
Source: Int J Mol Sci. 2024 Oct 27;25(21):11549. doi: 10.3390/ijms252111549 (PMC11546093; doi:10.3390/ijms252111549)
Supplement: Supplementary file 1 [file ijms-25-11549-s001.zip › ijms-3189387-supplementary figures.pdf]

Supplementary Figure S1

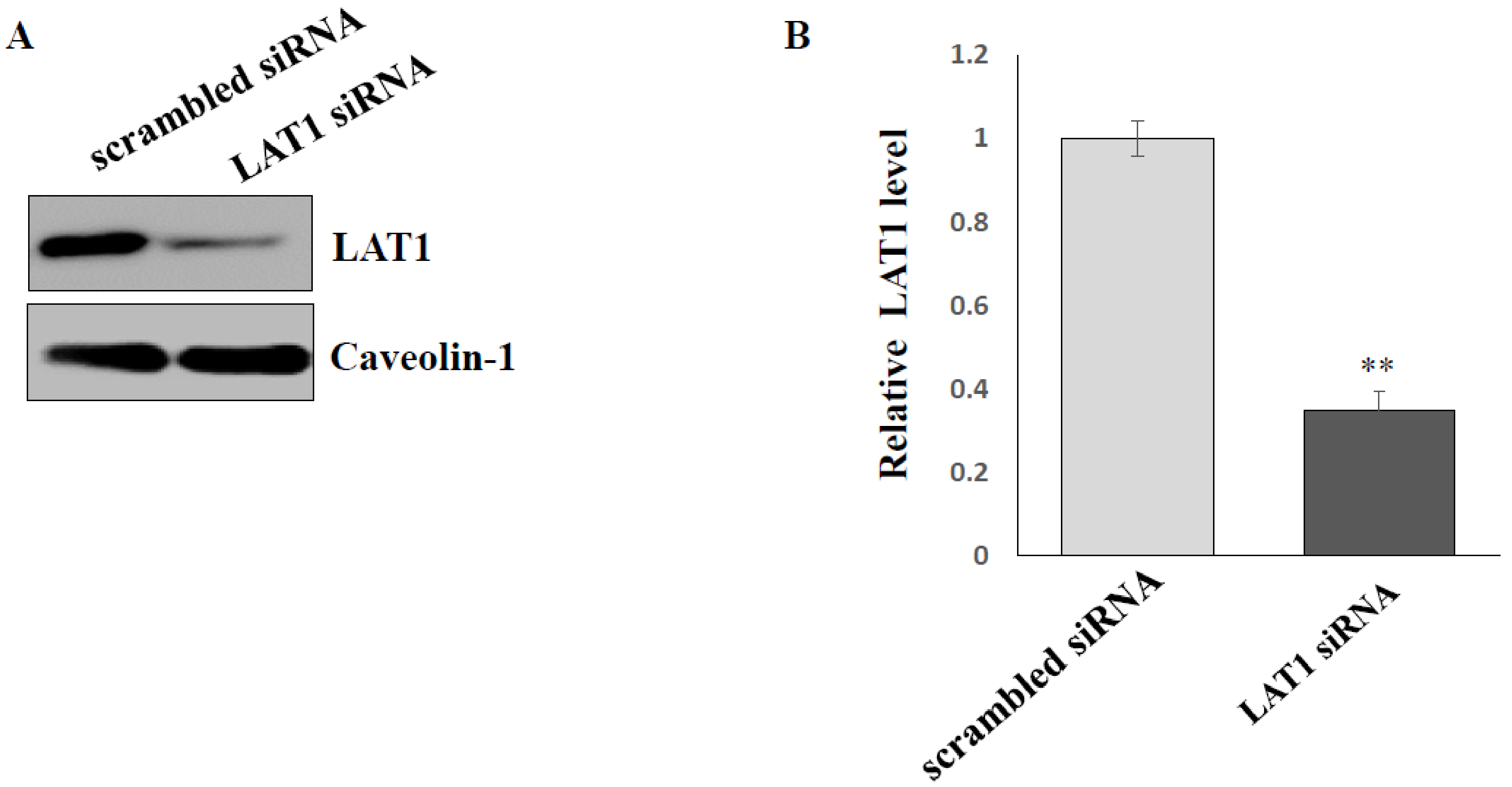

Supplementary Figure S1. (A) Representative images and (B) the quantitative data of LAT1 detected by immunoblots in C2C12 myotubes treated by scrambled or LAT1 siRNA. Results were normalized to the loading control and then compared the normalized data to scrambled siRNA. Data represent means ± SEM (*N*=3). ** P<0.01 compared to the scrambled siRNA group.

Supplementary Figure S2

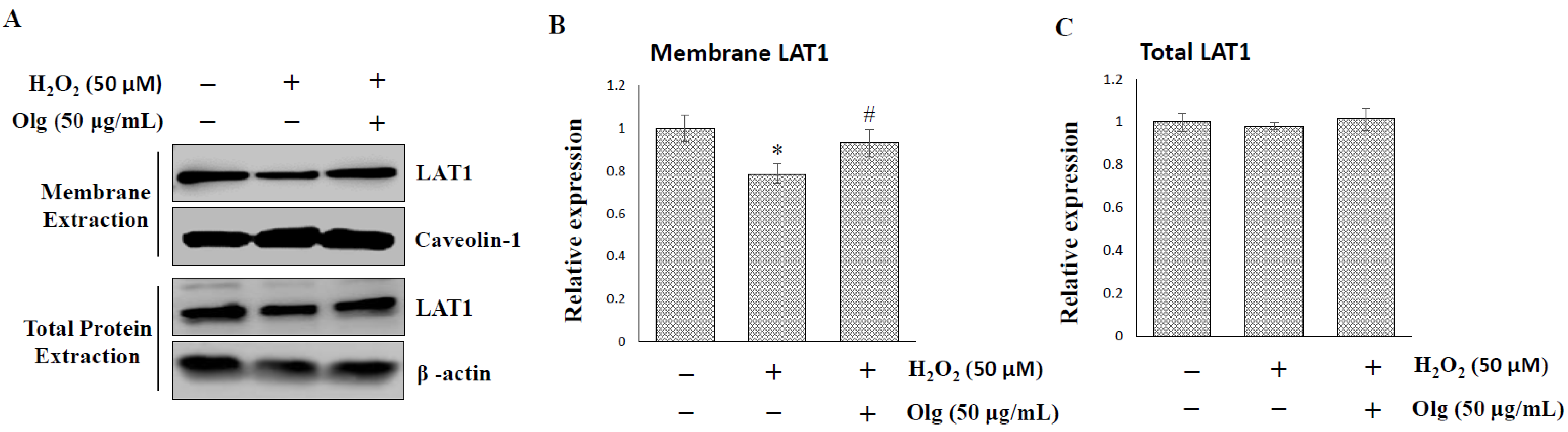

Supplementary Figure S2. (A) Representative images of LAT1 detected by immunoblots in C2C12 myotubes treated by H2O2. The quantification of LAT1 on (B) sarcolemma and (C) total lysate. Results were normalized to the loading control, and then the mean ratio to the untreated group was shown in the histograms. Data represent means ± SEM (*N*=4). * P<0.05 compared to control group. # P<0.05 compared to H2O2 treated group.

Supplementary Figure S3

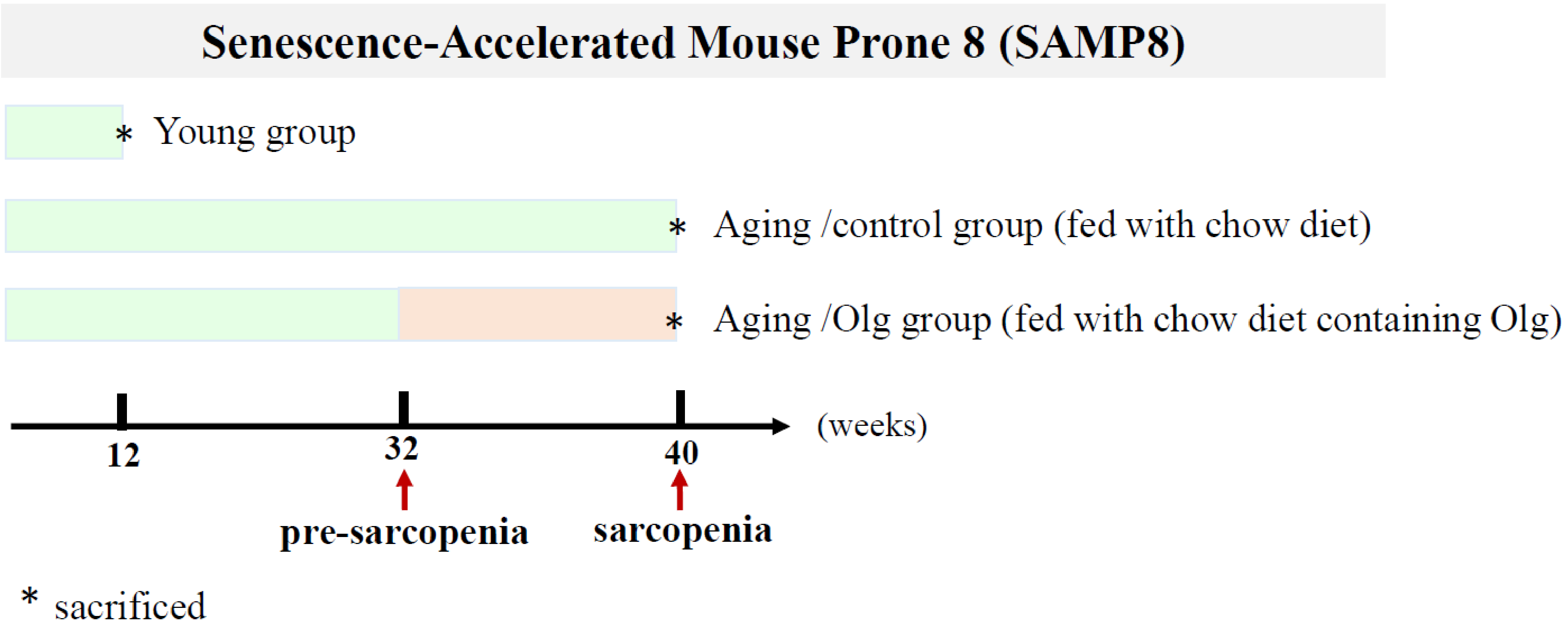

Supplementary Figure S3. Schematic diagram of experiments using a mouse model of SAMP8 mice.
