# Supplementary material for: Oligonol®, an Oligomerized Polyphenol from Litchi chinensis, Enhances Branched-Chain Amino Acid Transportation and Catabolism to Alleviate Sarcopenia
Source: Int J Mol Sci. 2024 Oct 27;25(21):11549. doi: 10.3390/ijms252111549 (PMC11546093; doi:10.3390/ijms252111549)
Supplement: Supplementary file 1 [file ijms-25-11549-s001.zip › ijms-3189387-supplementary table.pdf]

Supplementary Table S1. List of antibodies used in the studies

Primary antibody	Supplier	Host	Clonality	Dilution	Catalog
Anti-IgG	abcam	Rabbit	Monoclonal	1:2000	ab133470
Anti-Ubiquitin	abcam	Rabbit	Monoclonal	1:500	ab140601
LAT1	Novus Biologicals	Rabbit	Polyclonal	1:1000	NBP3-09988
mTOR	Cell Signaling	Rabbit	Monoclonal	1:1000	2983
Phospho-mTOR (Ser2448)	Cell Signaling	Rabbit	Monoclonal	1:1000	5536
p70S6K	Cell Signaling	Rabbit	Polyclonal	1:1000	9202
Phospho-p70S6K (Thr389)	Cell Signaling	Rabbit	Polyclonal	1:1000	9205
BCAT2	Cell Signaling	Rabbit	Monoclonal	1:1000	79764
beta Actin	abcam	Rabbit	Monoclonal	1:2000	ab115777
Caveolin-1	Cell Signaling	Rabbit	Monoclonal	1:1000	3267
GAPDH	GeneTex	Rabbit	Polyclonal	1:10000	GTX100118
Secondary antibody			Supplier	Dilution	Reference
Goat Anti-Rabbit IgG H&L (HRP)			abcam	1:5000	ab97051
Goat Anti-Rabbit IgG H&L (Alexa Fluor® 488)			abcam	1:1000	ab150077
